# BREAST-Q Under the Microscope: A Scoping Review of Reporting Gaps in Breast Surgery

**DOI:** 10.1093/asjof/ojag127

**Published:** 2026-06-23

**Authors:** Gabriela Fioranelli, Nicole Alvarez, Jace Boswell, Ton Doan, Leslie Christensen, Doruk Orgun, Aaron Dingle

## Abstract

The BREAST-Q was developed to address a gap in breast surgery outcomes research as a validated, procedure-specific patient-reported outcome measure (PROM) that assesses satisfaction and health related quality of life. Despite global adoption, reporting practices remain inconsistent. This review evaluated how BREAST-Q domains are reported and compated outcomes between postmastectomy reconstruction and cosmetic breast surgery populations. Five databases (PubMed, MEDLINE, PsycINFO, Scopus, and Web of Science) were searched from January 1, 2009 to September 16, 2022. Of 6921 records, 307 studies met inclusion criteria. Eligible studies reported mean BREAST-Q scores (0-100 scale) in patients undergoing postmastectomy reconstruction, breast augmentation, or breast reduction. Studies were grouped as reconstruction (Group 1) or cosmetic (Group 2). Extracted data included publication year, country, sample size, BREAST-Q means ± standard deviation and domain coverage. Analyses were descriptive due to heterogeneity in design, populations, and follow-up The most frequently reported domains in both groups were psychosocial well-being, satisfaction with breasts, and sexual well-being (Group 1: 79.12%, 91.21%, and 77.29%; Group 2: 74.55%, 96.36%, and 83.64%). The aggregate global weighted mean BREAST-Q score was higher in Group 2 than in Group 1 (81.99 vs 74.49), Though these values represent descriptive estimates, not direct comparisons. Although widely used, BREAST-Q reporting is inconsistent, with disproportionate emphasis on select domains. This selective reporting introduces bias and limits comparability. Comprehensive reporting across all domains, or inclusion of an overall summary score, would better reflect patient satisfaction and align with the instrument's intended purpose.

Level of Evidence: 3 (Therapeutic)

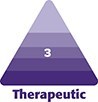

The BREAST-Q is a validated patient-reported outcome measure (PROM) developed to evaluate outcomes from the patient perspective among women undergoing breast surgery. It is administered as a self-completed questionnaire and was designed to capture patients' perceptions of surgical results and health-related quality of life following plastic and reconstructive breast procedures. Patient-reported outcomes have gained attention more recently as the surgical community attempts to move past traditional outcome measures such as morbidity and mortality and implement patient perception.^1^ Because patient satisfaction and quality of life are central indicators of success in both reconstructive and cosmetic breast surgery, the BREAST-Q was developed to address limitations of clinician-reported outcomes and to better reflect patient experiences.^2^

The BREAST-Q conceptual framework is structured around 2 core domains: quality of life and satisfaction. Quality of life domains include physical well-being, psychosocial well-being, and sexual well-being, whereas satisfaction domains encompass satisfaction with breasts, satisfaction with outcomes, and satisfaction with care.^2,3^ These domains are assessed through 3 modules with scales related to their procedures: breast augmentation, breast reduction, and breast cancer. The breast cancer module includes patients undergoing mastectomy, breast reconstruction, and breast-conserving therapy (BCT), whereas the augmentation and reduction modules are designed for cosmetic breast surgery populations.^2^

Each module consists of independent scales, allowing investigators to select domains relevant to their study objectives.

Since its introduction, the BREAST-Q has been increasingly adopted in breast surgery research worldwide. The instrument has been translated into 55 languages, facilitating broader implementation and cross-cultural comparisons of patient-reported outcomes. Information regarding available translations and modules is publicly accessible through the BREAST-Q developer website (qportfolio.org).

As the BREAST-Q has become more widely implemented, its flexibility has allowed investigators to select specific modules, domains, and time points based on individual study objectives. Although this adaptability has supported broad uptake across diverse clinical contexts, it has also resulted in heterogeneity in how the instrument is applied and reported. Studies vary considerably in domain selection, reporting completeness, and analytic approach across BREAST-Q studies.^4^ This heterogeneity may complicate cross-study comparisons and limit the synthesis of patient-reported outcomes across surgical populations. Beyond differences in domain reporting, gaps also exist in how the BREAST-Q has been applied across different surgical populations. Previous reviews have primarily focused on breast cancer–related modules with limited evaluation of cosmetic breast surgery populations.^1^

Consequently, a comprehensive assessment that integrates both domain-level reporting patterns and standardized PROMs across cosmetic and reconstructive breast surgery populations is needed.

Given the breadth of the literature, the variability in reporting and the need to characterize how the BREAST-Q has been applied across different breast surgical populations, a scoping review was considered the most appropriate methodological approach. The objective of this scoping review is to examine how the BREAST-Q has been reported across the breast surgery literature and to compare patient-reported outcomes across surgical populations. Specifically, this review addresses the following questions: (1) how the BREAST-Q is reported across breast surgery studies, including which domains are most and least frequently assessed; (2) patterns of domain reporting among cosmetic vs reconstructive breast surgery groups; and (3) differences in patient-reported outcomes between postmastectomy breast reconstruction and cosmetic breast surgery populations. By synthesizing findings across procedures and study designs, this review seeks to provide a comprehensive overview of current BREAST-Q use and highlight areas for greater consistency and standardization in future research.

## METHODS

This scoping review was reported in accordance with the Preferred Reporting Items for Systematic Review Extension for Scoping Reviews (PRISMA-ScR).^5^

### Eligibility Criteria

#### BREAST-Q–Related Criteria

Studies were eligible if they reported patient-reported outcomes using the BREAST-Q instrument, were written in English, and were published between 2009 and September 16, 2022. The search was limited to 2009 because the BREAST-Q was introduced during this period, and studies published before this date would not have used the instrument. Included studies were required to report patient perspective numerical BREAST-Q results as mean scores on a standardized 0 to 100 scale, with or without standard deviations (SDs). Only studies using the breast reconstruction, breast augmentation, or breast reduction BREAST-Q modules were included. Studies were excluded if they did not report BREAST-Q outcomes or if results were presented using noncomparable formats, including *P*-values without corresponding mean scores, medians with interquartile ranges, Likert-scale responses, raw scores not transformed to a 0 to 100 scale, or regression and beta coefficients without descriptive BREAST-Q data. Articles reporting BREAST-Q results only in graphical form without extractable numerical values were also excluded.

#### Population Criteria

Eligible populations included patients undergoing postmastectomy breast reconstruction, defined as mastectomy followed by any type of reconstructive procedure, and patients undergoing cosmetic breast surgery, defined as breast augmentation or breast reduction.

Studies were excluded if they reported normative populations without surgical intervention, or focused primarily on nonbreast outcomes, such as abdominal donor-site health.

#### Timeframe Criteria

Studies were included if they reported postoperative BREAST-Q outcomes alone or postoperative outcomes measured at one or multiple time points (eg, 3 months, 6 months, or 1 year). Studies reporting only preoperative outcomes were excluded.

#### Type of Surgery

Studies focused exclusively on BCT or breast-conserving surgery (BCS) without mastectomy were excluded. However, studies that included BCT or BCS populations as a comparison group alongside postmastectomy reconstruction patients were eligible, provided that postmastectomy reconstruction outcomes were reported separately and met all inclusion criteria.

#### General Exclusions

Non-English language publications were excluded. Secondary research, including review articles, study protocols, responses or commentaries to other studies, and studies describing the development or validation of BREAST-Q modules, were also excluded.

### Information Sources and Search Strategy

The review team collaborated with a research librarian (L.C.) to develop and execute a comprehensive search of the literature, utilizing both controlled vocabulary and keywords related to PROM in breast surgery. A search was conducted in the following databases from January 1, 2009 to September 16, 2022: PubMed, MEDLINE (EBSCO), APA PsycINFO (EBSCO), Scopus, and Science Citation Index-Expanded and Emerging Sources Citation Index as a multifile search in Web of Science Core Collection (Clarivate). In PubMed, non-MEDLINE records were excluded from the search results; in Scopus, records were limited to Embase records using an inclusion filter. No publication type or language filters were applied to the search results. The full search strategies for all databases are provided in Appendix 1. Results were downloaded to EndNote (Clarivate) and underwent manual deduplication by LC using the method described by Bramer et al.^6^ Unique records were uploaded to Covidence (Veritas Health Information, Melbourne, Australia) for further screening and review by the study team.

### Selection Criteria

Titles and abstracts were independently screened by 2 reviewers (G.F., N.A.), with conflicts resolved by team consensus. Full-text review was performed independently by 2 reviewers (G.F., N.A.), with conflicts resolved by team consensus.

### Data Extraction

For data extraction, 2 reviewers (G.F. and N.A.) independently charted data using a standardized Excel spreadsheet. Extracted data were compared for consistency, and discrepancies were resolved through team consensus.

Following study selection, data were divided into 2 main groups: postmastectomy reconstruction (Group 1) and cosmetic breast surgery (Group 2).

Postmastectomy reconstruction studies were further categorized into: (1) single-group postmastectomy studies, (2) postoperative timing comparisons, (3) types of reconstruction, (4) timing of reconstruction, and (5) other postmastectomy analyses. The latter included comparisons based on mastectomy type, receipt of radiotherapy, mastectomy with and without reconstruction, reconstructive materials or techniques, patient age and weight, complications, and type of breast cancer surgery (BCT vs postmastectomy reconstruction).

Cosmetic breast surgery studies were divided into augmentation and reduction groups, each further subdivided into single-group studies, preoperative/postoperative comparisons, and other comparative analyses.

Extracted data included comparison group, number of participants, mean values and SDs of BREAST-Q domains (see Appendix 1).

In most studies, the number of participants was the same across domains; however, when small differences in domain-specific respondent counts were reported, a single overall respondent number was still used so that each study could contribute to the pooled domain analyses using a consistent denominator. To achieve that for those studies, the average number of respondents for each domain was calculated for the single overall respondent number. For instance, if a study reported 100 respondents for satisfaction with breasts and 80 for psychosocial well-being, a study-level sample size of 90 was applied for weighting purposes across domains within that study. For studies reporting different numbers of respondents across individual BREAST-Q domains, the average number of respondents was calculated and used for analysis. When studies did not explicitly report the number of BREAST-Q respondents but provided the total number of participants included in each study group, the reported group sample size was used, provided that no dropouts were described. In cases where studies reported only an overall number of BREAST-Q respondents without stratification by comparison group, the study was excluded from quantitative data extraction.

For data analysis, some studies included overlapping comparison group types (eg, autologous postoperative 6-months, autologous postoperative 12 months, implant-based reconstruction [IBR] postoperative 6 months, IBR postoperative 12 months). These studies were included in both relevant subgroup analyses and are marked with asterisks (***) in the Supplemental Material. If an article included data from 2 different subgroups and the data could be separated, each result was assigned only to its most relevant subgroup, even if the article itself appeared in more than 1 subgroup. For example, in an article reporting autologous, implant-based, and immediate and delayed reconstruction outcomes, the autologous and implant-based data were assigned to the type of reconstruction subgroup, whereas the immediate and delayed reconstruction data were assigned to the timing of reconstruction subgroup.

### Statistical Analysis

Charted data were organized in Excel and stratified by predefined groups and subgroups (see Supplemental Section 2.3). Statistical analyses were performed using R and RStudio. Descriptive summaries included publication year, country of origin, and number of included articles. All analyses were descriptive in nature. Included studies differed substantially in patient populations, study design, outcome assessment, and follow-up duration, no direct statistical comparisons between groups or subgroups were performed. Domain coverage was assessed by calculating the percentage of articles reporting each BREAST-Q domain and the percentage of patients contributing data to each domain. Within each group, we examined the 3 most commonly reported BREAST-Q domains: Psychosocial well-being, satisfaction with breasts, and sexual well-being, based on the percentage of studies reporting each domain. Weighted means and pooled SDs were calculated for each domain across relevant subgroups. Weighted means represent aggregated study-level estimates and do not reflect pooled patient-level data; therefore, they are presented for descriptive purposes only.

## RESULTS

### Study Selection

**Figure 1.**
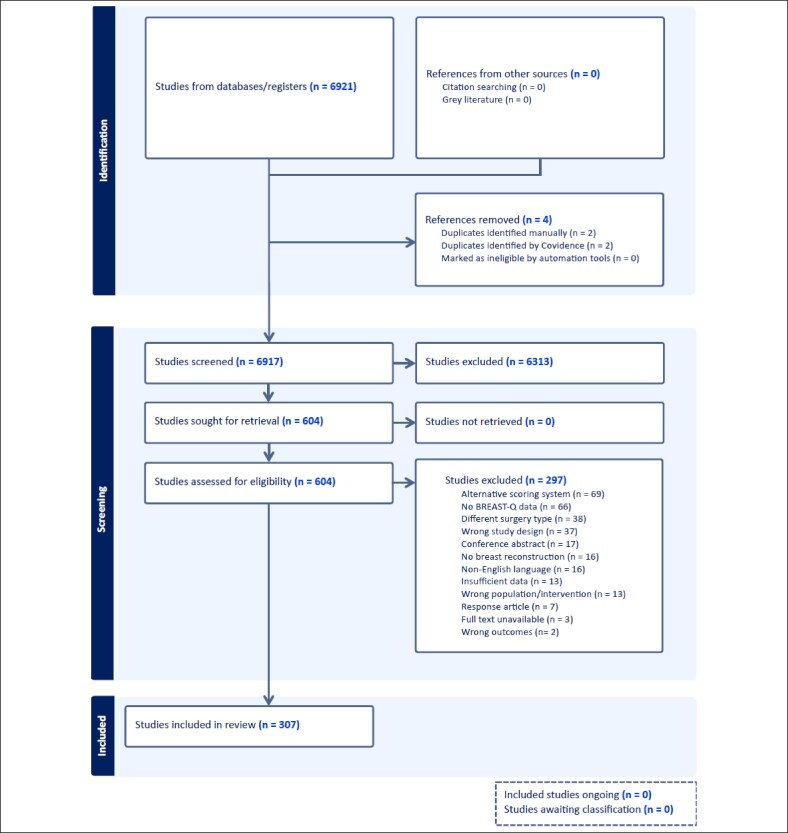
PRISMA flow.

The initial search identified 6921 unique records for title and abstract screening. Following title and abstract screening, 604 records underwent full-text review, of which 307 met the criteria for inclusion in the review (Figure 1). The BREAST-Q domains captured in the 307 studies are summarized in Table 1. For the full list of included articles, see Appendix 1.

**Table 1.** BREAST-Q Domains Extracted From Included Studies

Satisfaction with breasts	Satisfaction with information	Satisfaction with outcomes
Satisfaction with care	Satisfaction with surgeon	Satisfaction with nipples
Satisfaction with medical staff	Satisfaction with office staff	Psychosocial well-being
Psychosocial well-being	Physical well-being of the chest and upper body	Physical well-being of the abdomen

Data was extracted from the 307 studies with variables charted including type of surgery (eg, autologous, IBR, augmentation, reduction), country of origin, year of publication, number of participants, and the BREAST-Q domains assessed. The sample sizes ranged from 5 to 1306 participants. There was a strong representation from countries in North America, Europe and Asia. Studies from Africa were not identified, and South America was minimally represented, indicating a potential geographic gap in the current literature.

### BREAST-Q Reporting and Outcomes

#### Overview of Included Studies

The included studies were categorized into 2 main groups:

Group 1: postmastectomy reconstruction
Group 2: cosmetic breast surgery

### Group 1: Postmastectomy Reconstruction Group

#### Overall Domain Reporting

Satisfaction with breasts: reported in 91.21% of articles

Psychosocial well-being: reported in 79.12%

Sexual well-being: reported in 77.29%

#### Group Analysis

**Figure 2.**
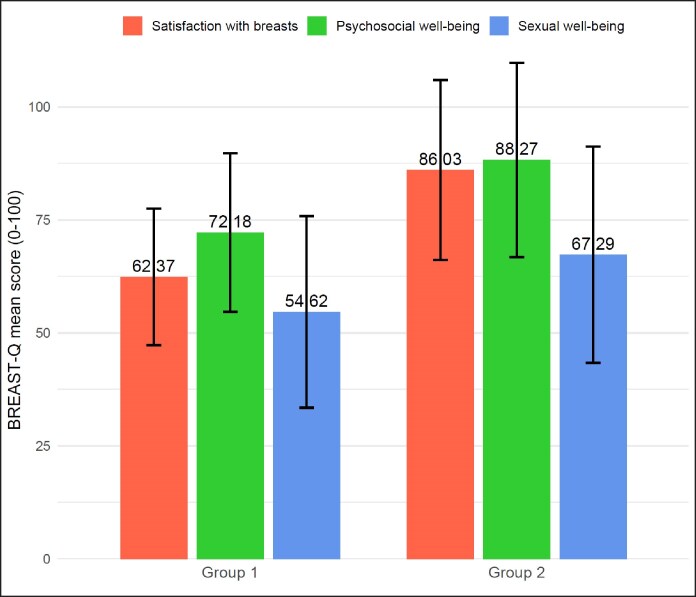
Weighted mean BREAST-Q scores and standard deviations for satisfaction with breasts, psychosocial well-being, and sexual well-being in Groups 1 and 2.

Overall, patients in the postmastectomy reconstruction group reported a mean satisfaction with breasts score of 62.37 ± 15.11, a mean psychosocial well-being score of 72.18 ± 17.52, and sexual well-being score of 54.62 ± 21.19 (Figure 2).

#### Subgroup Analyses

Overall domain reporting was further stratified according to study design subgroups (Table 2). These results are descriptive and intended to characterize reporting patterns and aggregated BREAST-Q outcomes across the literature. Because of substantial heterogeneity among included studies, these subgroup summaries should not be interpreted as direct comparative evaluation.

**Table 2.** Group 1 Weighted Mean Scores and Pooled Standard Deviations for Psychosocial Well-Being, Satisfaction With Breasts, and Sexual Well-Being Domains in Patients Undergoing Postmastectomy Breast Reconstruction, Stratified by Study Design Subgroup

Subgroup	No. of articles	Psychosocial well-being (mean ± SD)	Satisfaction with breasts (mean ± SD)	Sexual well-being (mean ± SD)
Single group	61	74.45 ± 20.37	66.08 ± 19.87	55.14 ± 23.99
Postoperative timing	37	74.62 ± 19.69	63.99 ± 17.78	54.64 ± 22.26
Type of reconstruction	75	72.13 ± 19.88	66.01 ± 17.88	54.21 ± 22.21
Timing of reconstruction	28	73.79 ± 19.41	65.77 ± 18.21	55.48 ± 22.41
Others	111	64.62 ± 17.29	59.97 ± 14.68	69.92 ± 18.73

SD, standard deviation.

### Group 2: Cosmetic Breast Surgery Group

#### Overall Domain Reporting

Satisfaction with breasts: 96.35% of studies

Psychosocial well-being: 74.55%

Sexual well-being: 83.64%

#### Group Analysis

Patients in the cosmetic breast surgery group reported a pooled average of 86.03 ± 19.9 for satisfaction with breasts, 88.27 ± 21.49 for psychosocial well-being, and 67.29 ± 23.9 for sexual well-being (Figure 2).

#### Subgroup Analyses

Overall domain reporting was further stratified, firstly by operating type; breast augmentation (Table 3) and breast reduction (Table 4), each of which are further stratified according to study design subgroups. These results are descriptive and intended to characterize reporting patterns and aggregated BREAST-Q outcomes across the literature. Because of substantial heterogeneity among included studies, these subgroup summaries should not be interpreted as direct comparative evaluations.

**Table 3.** Group 2 (Augmentation), Weighted Mean Scores and Pooled Standard Deviations for Psychosocial Well-Being, Satisfaction With Breasts, and Sexual Well-Being Domains in Patients Undergoing Breast Augmentation, Stratified by Study Design Subgroup (Single Group, Postoperative Timing, and Other)

Augmentation				
Subgroup	No. of articles	Psychosocial well-being (mean ± SD)	Satisfaction with breasts (mean ± SD)	Sexual well-being (mean ± SD)
Single group	21	74.28 ± 21.00	75.49 ± 20.45	73.71 ± 23.32
Postoperative timing	5	89.46 ± 18.06	87.82 ± 15.31	82.35 ± 19.96
Others	11	89.29 ± 21.93	87.74 ± 17.77	66.05 ± 20.76

SD, standard deviation.

**Table 4.** Group 2 (Reduction), Weighted Mean Scores and Pooled Standard Deviations for Psychosocial Well-being, Satisfaction With Breasts, and Sexual Well-being Domains in Patients Undergoing Breast Reduction, Stratified by Study Design Subgroup (Single Group, Postoperative Timing, and Other)

Reduction				
Subgroup	No. of articles	Psychosocial well-being (mean ± SD)	Satisfaction with breasts (mean ± SD)	Sexual well-being (mean ± SD)
Single group	10	76.65 ± 21.60	73.29 ± 19.58	68.53 ± 22.67
Postoperative timing	2	81.24 ± 24.61	80.72 ± 25.11	75.78 ± 30.54
Others	7	76.7 ± 21.08	69 ± 19.6	70.92 ± 23.75

SD, standard deviation.

### Domain Variability by Groups and Subgroups

Across the main groups (Groups 1 and 2), the most frequently reported BREAST-Q domains (Table 1, Appendix 1) were psychosocial well-being, satisfaction with breasts, and sexual well-being. There was variability within specific subgroups, particularly those with a lower number of studies. In the “breast augmentation—post op timing” subgroup (*n* = 5 articles), 100% of studies reported both Satisfaction with Breasts and Psychosocial Well-Being, whereas Physical Well-Being and Sexual Well-being were each reported in 80% of articles. In the “breast augmentation—others” subgroup (n = 11), Satisfaction with Breasts was reported in 90.91% of articles, Satisfaction with Outcome and Psychosocial Well-Being in 81.82% of articles, and Sexual Well-Being in 72.73%. In the “breast reduction—post op timing” subgroup (*n* = 2 articles), all studies (100%) reported Physical Well-being, Psychosocial Well-being, Satisfaction with Breasts, and Sexual Well-being. In the “breast reduction—others” subgroup (*n* = 7 articles), Satisfaction with Breasts was consistently reported in 100% of studies, whereas Physical Well-being, Psychosocial Well-being, and Sexual Well-being were each reported in 85.71% of studies. Lastly, in the “breast reduction—single” subgroup (*n* = 10 articles), all studies (100%) reported Satisfaction with Breasts, whereas Physical Well-being, Psychosocial Well-being, and Sexual Well-being were each reported in 90% of articles.

### Multi-Subgroup Studies

A total of 43 studies reported outcomes across 2 distinct subgroups and 2 studies included data from 3 different subgroups.

### Summary of Pooled BREAST-Q Scores

**Figure 3.**
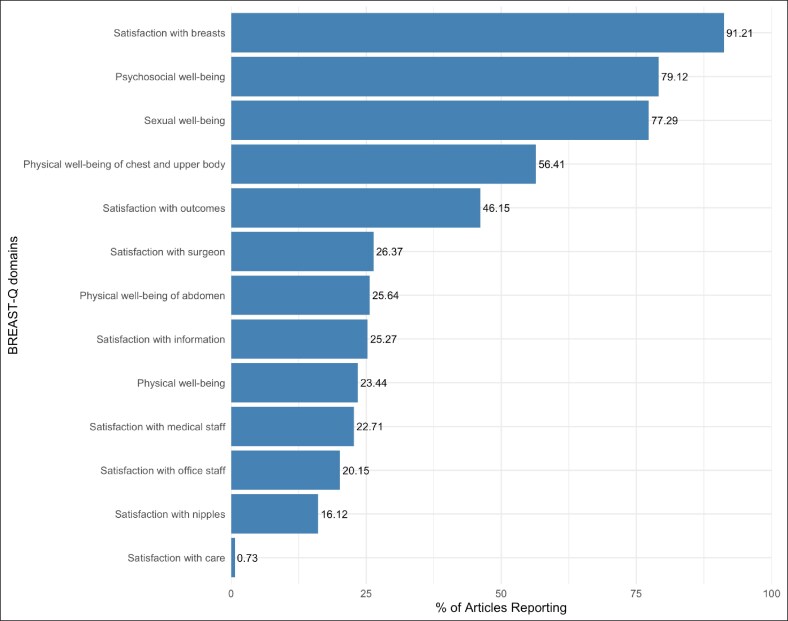
Percentage of articles reporting each BREAST-Q domain in Group 1.

**Figure 4.**
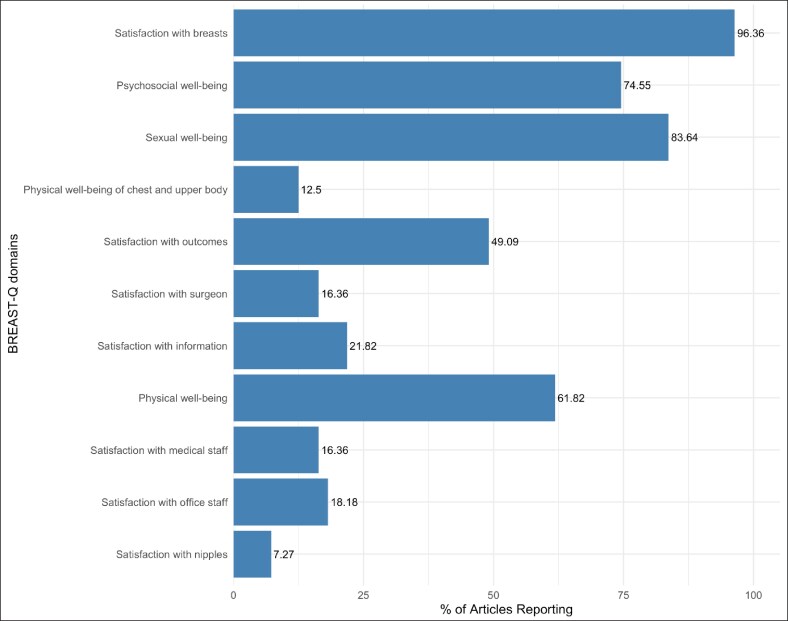
Percentage of articles reporting each BREAST-Q domain in Group 2.

The aggregate global weighted mean BREAST-Q score for Group 2 was 81.99, and for Group 1 74.49. Both main groups included responses across domains: physical well-being, physical well-being of chest, psychosocial well-being, satisfaction with breasts, satisfaction with information, satisfaction with medical staff, satisfaction with nipples, satisfaction with office staff, satisfaction with outcomes, satisfaction with surgeon, and sexual well-being. The postmastectomy reconstruction group additionally included data for physical well-being of the abdomen and satisfaction with care. Percentages of domains reported for each group are provided in Figures 3, 4.

Within the postmastectomy reconstruction group (Table 2), subgroup level mean scores including all domains were: “postoperative timing” 76.51, “single group” 76.93, “timing of reconstruction” 76.88, “type of reconstruction” 76.79, and “others” 72.97.

In the cosmetic surgery group, breast augmentation (Table 3) and breast reduction (Table 4) subgroups also exhibited variation. Among breast augmentation subgroups, aggregated global mean scores were: “single group” 81.03, “others” 84.13, and “postoperative timing” 83.69. For breast reduction subgroups, scores were: “others” 81.57, “single group” 75.63, and “post op timing” 80.69.

Complete domain-level data, including weighted mean BREAST-Q scores, pooled SDs, number of articles reporting each domain, number of patients contributing to each domain, and percentages of articles and patients reporting each domain, are provided in Appendix 2 for Group 1 and Appendix 3 for Group 2.

### Synthesis of Results

Analysis of the extracted data across 307 included studies revealed that psychosocial well-being, satisfaction with breasts, and sexual well-being were the most commonly reported BREAST-Q domains in both the postmastectomy reconstruction and cosmetic breast surgery groups. Consistent with the aims of this review, these domains were selected for pooled analysis because of their frequency and clinical relevance. Descriptive aggregated BREAST-Q scores reported in studies of cosmetic breast surgery populations were numerically higher than those reported in studies of postmastectomy reconstruction populations across the 3 most frequently reported domains, including psychosocial well-being (88.27 ± 21.49 vs 72.18 ± 17.52), satisfaction with breasts (86.03 ± 19.9 vs 62.37 ± 15.11), and sexual well-being (67.29 ± 23.90 vs 54.62 ± 21.19). These values represent aggregated study-level estimates and are presented descriptively rather than as direct statistical comparisons.

The least frequently reported domains were satisfaction with nipples (16.12% in postmastectomy group; 7.27% in cosmetic group), satisfaction with office staff (20.15%; 18.18%), and satisfaction with surgeon (26.37%; 16.36%).

The aggregate weighted mean across reported BREAST-Q domains score was numerically higher in Group 2 (81.99) compared with Group 1 (74.49), reflecting overall trends in patient-reported outcomes across the included studies.

## DISCUSSION

### Summary of Evidence

#### Methodological and Reporting Heterogeneity in the BREAST-Q Literature

The BREAST-Q was developed to address a critical gap in breast surgery outcomes research by capturing patient-reported quality of life and satisfaction across physical, psychosocial, and sexual well-being domains, as well as satisfaction with breasts, outcomes, and care, using procedure-specific modules for augmentation, reduction, and breast cancer populations.^2^ As outlined in the BREAST-Q User's Guide, its modular design permits investigators to administer only those scales most relevant to a given patient population or research question. Although this flexibility has facilitated widespread adoption since the instrument's introduction in 2009, it has also contributed to substantial variability in how domains and modules are selected, applied, and reported across the literature.

Consistent with previous work, the present scoping review demonstrates that BREAST-Q utilization remains uneven across postmastectomy reconstruction and cosmetic breast surgery populations. Previous systematic reviews have largely focused on breast cancer–related modules, with comparatively limited evaluation of cosmetic breast surgery populations.^7,8^ Our findings quantitatively confirm this imbalance: among the 307 included studies, 255 evaluated postmastectomy reconstruction and only 34 breast augmentation and 18 breast reduction studies met inclusion criteria. This disproportionate representation parallels observations by Cohen et al, who reported that the reconstruction module is the most frequently utilized, likely reflecting sustained academic focus on breast cancer.^1^ However, this emphasis contrasts with contemporary procedural volume data, in which cosmetic breast surgeries substantially outnumber reconstructions, suggesting that patient-reported outcomes in cosmetic populations remain underrepresented within the BREAST-Q literature.

Cosmetic underrepresentation is likely because of increased motivation to provide evidence-based care in cancer care vs cosmetic. Advocacy efforts like the push for the Women's Health Care and Cancer Care Act demonstrate the priority given to cancer care and research on postmastectomy patients using the BREAST-Q further underscore the importance of these efforts.^9^ Increased research on cosmetic patients using the BREAST-Q may help characterize deficits in patient quality of life that can be addressed and help to create better and more comprehensive care for all breast patients.

#### Patterns of Domain Reporting Across Surgical Populations

Beyond disparities in module utilization, domain-level reporting was also inconsistent. Across both postmastectomy reconstruction (Figure 3) and cosmetic (Figure 4) breast surgery groups, satisfaction with breasts, psychosocial well-being, and sexual well-being were the most frequently reported BREAST-Q domains. Satisfaction with breasts was the most commonly reported domain overall, appearing in 86.7% of postmastectomy reconstruction studies and 88.52% of cosmetic breast surgery studies, consistent with previous observations that investigators often prioritize satisfaction with breasts as a primary endpoint.^1^

In contrast, other domains integral to the BREAST-Q's multidimensional framework, including satisfaction with care, satisfaction with staff, and nipple satisfaction were infrequently reported. Sexual well-being was the lowest-scoring domain overall and, despite being reported in 83.64% of cosmetic studies, was represented in only 14.96% of patients in the cosmetic group. This finding likely reflects the fact that sexual well-being is influenced by a broad range of factors beyond breast appearance, including relationship dynamics, body image, emotional health, partner support, and overall psychosocial context. In postmastectomy populations, these scores may also be shaped by the psychological burden of a cancer diagnosis and its effect on both the patient and their partner, making sexual well-being a more complex outcome that may not improve with breast satisfaction alone.

Interpretation is further limited by the fact that not all studies reported preoperative and postoperative comparisons; many presented only a single postoperative score, which makes it difficult to determine the degree of change attributable to surgery itself. The limited reporting of this specific domain may also reflect persistent stigma or discomfort surrounding sexual concerns in both research and clinical settings. More broadly, underutilization of these domains may reflect study design constraints, selective reporting practices, or challenges in assessment, but ultimately limits comprehensive characterization of patient experience and quality-of-life outcomes following breast surgery.

#### Descriptive Patterns Across Cosmetic and Reconstructive Breast Surgery Studies

Studies involving cosmetic breast surgery populations reported numerically higher aggregated psychosocial well-being scores than studies involving postmastectomy reconstruction populations (Figure 2). These findings likely reflect differences in clinical context, baseline health status, and patient expectations between populations. Patients undergoing postmastectomy reconstruction frequently contend with cancer diagnosis, adjuvant therapies, and loss of native breast tissue, all of which may influence postoperative quality of life and satisfaction. In contrast, cosmetic breast surgery patients typically undergo elective procedures in the absence of malignancy.

These results emphasize the importance of interpreting BREAST-Q scores within their appropriate clinical context rather than as absolute measures of surgical success.

### Limitations

#### Methodological and Reporting Limitations Impacting Secondary Synthesis

A major challenge identified in this review was substantial heterogeneity in BREAST-Q reporting practices, even among studies using the same module. Several studies reported only a single BREAST-Q domain, while omitting other core quality-of-life and satisfaction domains. In other cases, key domains such as satisfaction with breasts, psychosocial well-being, or sexual well-being were reported using noncomparable formats (eg, medians with interquartile ranges, percentages, or graphical displays) rather than standardized mean scores, precluding inclusion in pooled quantitative analyses. Such selective and inconsistent reporting undermines the multidimensional intent of the BREAST-Q instrument and limits secondary synthesis. Consequently, apparent differences between subgroup estimates should not be interpreted as evidence of superiority or inferiority of any procedure, technique, or patient population.

Some articles reported discrepant BREAST-Q values between text and tables for the same domain, while others presented domain-level results stratified by clinical subgroups (eg, ptosis or reconstruction type) without providing overall cohort-level means. For consistency, this review relied on aggregated cohort-level scores when available, though this approach may obscure clinically meaningful subgroup differences.

#### Challenges Related to Scale Selection, Terminology, and Respondent Reporting

Additional complexity arose from variation in scale selection and domain labeling. Several studies reported procedure-specific or optional scales, such as satisfaction with implants, donor-site well-being, implant rippling, or flap donor-site appearance, that are not uniformly applicable across surgical populations or consistently reported on the standard 0 to 100 BREAST-Q scale. While such scales may provide valuable procedure-specific insight, their inconsistent use limited inclusion in pooled analyses.

Moreover, some studies used BREAST-Q terminology imprecisely, referring to constructs such as “satisfaction with breast appearance” and later reporting only a general “Satisfaction rate,” without explicitly identifying the validated BREAST-Q domain assessed. This ambiguity may lead to misinterpretation and limits comparability.

#### Methodological Limitations of the Review Process

Several limitations should be considered when interpreting the results of this study. Firstly, this review explicitly utilized BREAST-Q reports that were presented in published literature rather than raw survey data. As such, it is not possible to infer whether authors collected certain domain scores or whether the data was selectively presented to show only favorable results, potentially introducing reporting bias. Therefore, the reasoning behind exclusion of this information cannot be definitively established. Also, patients with favorable results may have been motivated to follow up and complete the BREAST-Q, possibly contributing to both selection bias as well as attrition bias in those who chose not to complete the BREAST-Q after being dissatisfied with results. Another limitation that should be considered is potential domain misclassification because of ambiguous or nonstandardized terminologies used within studies, making it difficult to determine data completeness. Although standardized extraction protocols were applied, reviewer interpretation of domain reporting introduces potential for extraction bias, emphasizing the importance of maintaining consistent wording in order to enhance cross-study comparability and limit misleading exclusion of relevant data. Incomplete reporting of the number of patients contributing to BREAST-Q data was also a recurrent limitation. Many studies reported overall surgical sample sizes without specifying the number of respondents completing the BREAST-Q overall, by subgroup, or at specific time points. In longitudinal designs, attrition was often reported only as aggregate percentages rather than group-specific counts, making accurate determination of respondent numbers difficult. As a result, assumptions were sometimes required or studies excluded from quantitative synthesis despite otherwise relevant clinical data. Several studies also only clearly listed one or few domains and presented the remaining domain scores in graphs without exact numbers, rather than providing a comprehensive table with all collected BREAST-Q scores quantified. In addition, only studies published in English were included, potentially excluding pertinent data published in other languages. Lastly, both preoperative and postoperative BREAST-Q data were included when reported, and aggregation of these time points constrained evaluation of change in scores over time. Future studies would benefit from implementing standardized reporting of BREAST-Q outcomes by time point with domain-specific respondent counts to improve transparency and interpretability in secondary analyses.

### Clinical Implications and Standardized Recommendations for BREAST-Q Reporting

**Figure 5.**
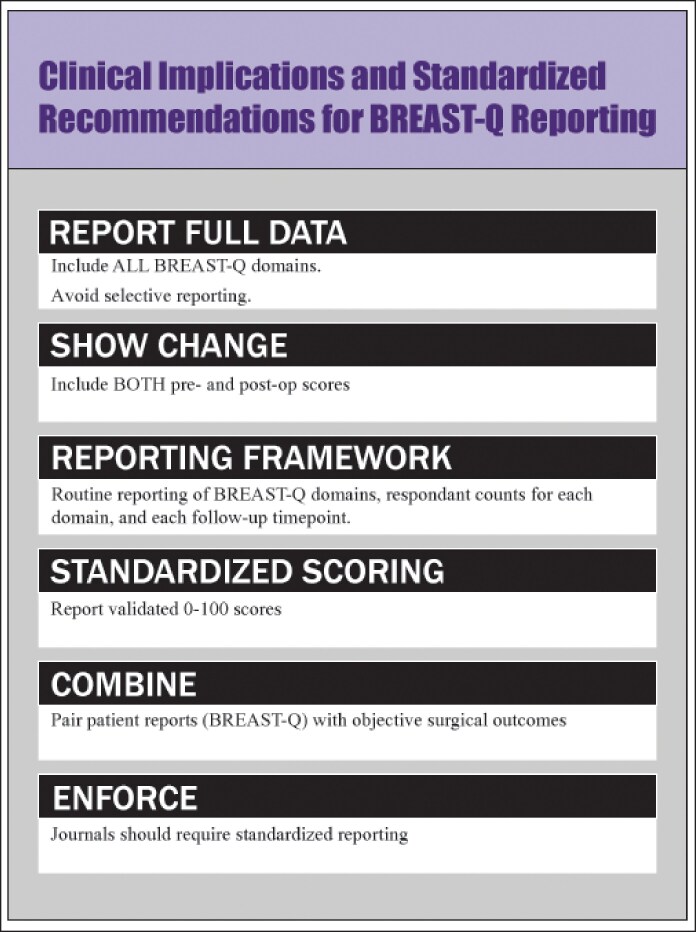
Summary of key recommendations for standardized BREAST-Q reporting.

The findings of this review have several practical implications for clinicians, researchers and journal editors (Figure 5). First, investigators should recognize that the selective reporting of BREAST-Q domains is common. The BREAST-Q was designed as a multidimensional instrument and reporting only selected domains risks overlooking important aspects of quality of life.

Second, it is important that both preoperative and postoperative scores are presented wherever possible, as this ensures quantifiable change attributed to the surgery itself.

Third, a minimum reporting framework may help facilitate consistency across studies. Future breast surgery studies should routinely report Satisfaction with Breasts, Psychosocial Well-Being, Sexual Well-Being, and Physical Well-Being. Reporting respondent counts for each domain and each follow-up time point should also be encouraged to improve transparency and facilitate future evidence synthesis.

Fourth, greater standardization of BREAST-Q reporting would improve cross-study comparability. Consistent use of validated 0-100 transformed scores, explicit identification of domains assessed, and reporting of all collected domains would facilitate interpretation by clinicians and allow more meaningful aggregation of outcomes across studies.

Fifth, where possible, validated, objective surgical scoring systems should be reported in conjunction with BREAST-Q data to better identify and understand discrepancies between patient perception and measurable aesthetic or surgical outcomes.

Finally, journal editors and reviewers play an important role in promoting comprehensive BREAST-Q reporting. Development and enforcement of a standardized BREAST-Q reporting framework could reduce heterogeneity and improve the quality of patient-reported outcomes research in breast surgery.

## CONCLUSIONS

This scoping review demonstrates that despite widespread adoption of the BREAST-Q, its application across breast surgery research remains uneven and inconsistently reported. Postmastectomy reconstruction is overrepresented relative to cosmetic breast surgery, and domain-level reporting frequently prioritizes satisfaction with breasts, psychosocial and sexual well-being while underutilizing other core quality-of-life and satisfaction domains. Descriptive aggregate BREAST-Q scores were numerically higher among studies evaluating cosmetic breast surgery populations than among studies evaluating postmastectomy populations; however, these findings should not be interpreted as direct comparative evidence because no statistical comparisons were performed.

Future studies should adopt standardized reporting practices, including transparent reporting of respondent numbers, consistent use of validated domain terminology, and comprehensive reporting of all collected BREAST-Q domains to improve comparability and maximize the clinical utility of patient-reported outcomes research.

## Supplementary Material

ojag127_Supplementary_Data
